# A Plant-Based Animal Fat Analog Produced by an Emulsion Gel of Alginate and Pea Protein

**DOI:** 10.3390/gels9050393

**Published:** 2023-05-09

**Authors:** Chong Teng, Osvaldo H. Campanella

**Affiliations:** Department of Food Science and Technology, Ohio State University, 2015 Fyffe Road, Columbus, OH 43210, USA; teng.165@buckeyemail.osu.edu

**Keywords:** pea protein, alginate, plant-based fat analog, DSC, FTIR, rheometry, colorimetry

## Abstract

As the market for plant-based meat analogs grows, the development of plant-based animal fat analogs has become increasingly important. In this study, we propose an approach by developing a gelled emulsion based on sodium alginate, soybean oil (SO), and pea protein isolate. Formulations containing 15% to 70% (*w*/*w*) SO were successfully produced without phase inversion. The addition of more SO resulted in pre-gelled emulsions with a more elastic behavior. After the emulsion was gelled in the presence of calcium, the color of the gelled emulsion changed to light yellow, and the formulation containing 70% SO exhibited a color most similar to actual beef fat trimming. The lightness and yellowness values were greatly influenced by the concentrations of both SO and pea protein. Microscopic images revealed that pea protein formed an interfacial film around the oil droplets, and the oil was more tightly packed at higher oil concentrations. Differential scanning calorimetry showed that lipid crystallization of the gelled SO was influenced by the confinement of the alginate gelation, but the melting behavior was like that of free SO. FTIR spectrum analysis indicated a potential interaction between alginate and pea protein, but the functional groups of SO were unchanged. Under mild heating conditions, gelled SO exhibited an oil loss similar to that observed in actual beef trims. The developed product has the potential to mimic the appearance and slow-rendering melting attribute of real animal fat.

## 1. Introduction

There has been a 58% increase in the worldwide demand for meat in the past twenty years due to the rising global population and the expanding economy [1]. The demand for protein is expected to continue to rise in tandem with population growth. However, the production of meat is associated with massive land and water demand, high volumes of greenhouse gas emissions, risks of animal diseases, etc. [2]. Moreover, the consumption of meat has been shown to have effects on human health, such as augmented risks of colorectal cancer and gastritis, among other cancer or non-cancer illness [3,4]. Plant-based meat analogs have been created to mimic the texture and flavor of real meat products and meat analog consumers in Europe and North America are currently meat eaters [5]. Environmental concerns have been driving consumers to move to a plant-based diet and reject the killing of animals to procure their food.

Two major components of plant-based meat analogs are proteins and lipids. Most manufacturers adopt extrusion cooking to convert globular plant-based proteins into meaty fibrous structures. Aside from proteins, the presence of lipids is also of essential importance, as they provide juiciness, tenderness, and various flavors to the products. One of the major challenges in developing an animal fat analog is to simulate the slow-rendering behavior of animal adipose tissues using vegetable materials. A significant portion of plant-based meat analog manufacturers now rely heavily on the direct addition of coconut oil in plant-based meat analogs because, like animal fat, it is solid at room temperature. Although the association between saturated fatty acid (SFA) content and risks of heart disease may be debatable [6,7], coconut oil’s high SFA content and rapid melting at high temperatures hinder its wide application and consumer acceptability. In this sense, plant oil abundant in unsaturated fatty acids has gathered more research attention. We have chosen soybean oil as our material because it is a well-studied and globally consumed vegetable oil for its beneficial content of tocopherol, polyunsaturated fatty acids (PUFAs), absence of trans-fat, and high heat tolerance. However, its liquid feature at room temperature is unfavorable, since it will readily separate from the food matrix without providing the desired adipose-like texture provided by the real fat trim.

Recent research has proposed approaches to structure plant oils. For example, temperature-sensitive oleogelators, such as waxes, monoglycerides, ethylcellulose, etc., were mixed with liquid oil at high temperatures and the system was cooled to form an elastic material. Glycerol monolaurate was used as an oleogelator to form an oleogel having higher heat stability and a lower oxidation rate [8]. Nonetheless, concerns on waxy mouthfeel, high cost, incompatibility with different oils, and the use of high amounts of oleogelators have still been limiting factors for wide applications [8,9,10]. Recently, gelled emulsion have emerged as promising solutions to produce analog fat trims. They are not only able to accurately replicate the physical properties of animal fats, such as water holding capacity and hardness, but they may also may preserve lipids and flavors from deleterious reactions [11]. For example, corn oil was emulsified with different types of proteins and gelled by glucono-δ-lactone, inducing protein crosslinking. Even at an oil concentration of 20%, oil droplet aggregation and creaming occurred [12]. A recent study showed the possibility of using agar to form gelled emulsions; however, the gel was too soft compared to real animal adipose tissue [13].

In this study, we aimed to entrap soybean oil using pea protein as the emulsifier and sodium alginate as the gelling agent. Sodium alginate is a polysaccharide derived from marine brown algae (*Phaeophyceae*) that is composed of 1,4-β-d-mannuronic (M) and α-l-guluronic (G) acids. The G units can be crosslinked by divalent calcium ions, which results in a thermal, irreversible, yet pH-sensitive gel [14], making it an ideal carrier for the controlled release of encapsulated compounds [15]. However, sodium alginate alone is in most cases used for encapsulating hydrophilic bioactive compounds, as creaming or sedimentation could readily occur if hydrophobic compounds are chosen as the filler. Therefore, a proper emulsifier is required to homogenize and stabilize the mixture. A mixture of Tween 80 and Span 20 has been used to steadily encapsulate a high portion of oil [16], but the thermal behavior of the product was not discussed. Other attempts have used protein as the emulsifier; however, the proteins were mostly animal-derived or the oil load was low [17,18,19]. Our previous results showed that the presence of the small amount of native protein present in butter was able to form and stabilize a gelled butter emulsion with a high portion of lipids. In the present study, we focused on constructing the gelled oil using completely plant-derived materials. Pea protein was selected as the emulsifier, as it is evidenced to have good nutritional quality and a better emulsifying capacity while having low allergenicity to humans [20,21]. Pea protein is also used as a suitable protein to produce the texturization necessary to provide meat analogs [22].

A heat-stable emulsion gel that simulates the texture, appearance, and heat behavior of animal fat trim was developed in the study. A sodium alginate solution and soybean oil were emulsified using pea protein isolate as the emulsifier. The pre-emulsion was gelled in the presence of calcium. The influence of the oil content on the rheology of the emulsion, color attribute, functional group changes, microstructure, and thermal stability of the gelled emulsion were studied. The product is completely prepared with plant-derived compounds and could potentially be scaled up for industrial manufacturing.

## 2. Results and Discussion

### 2.1. Rheology

**Figure 1:**
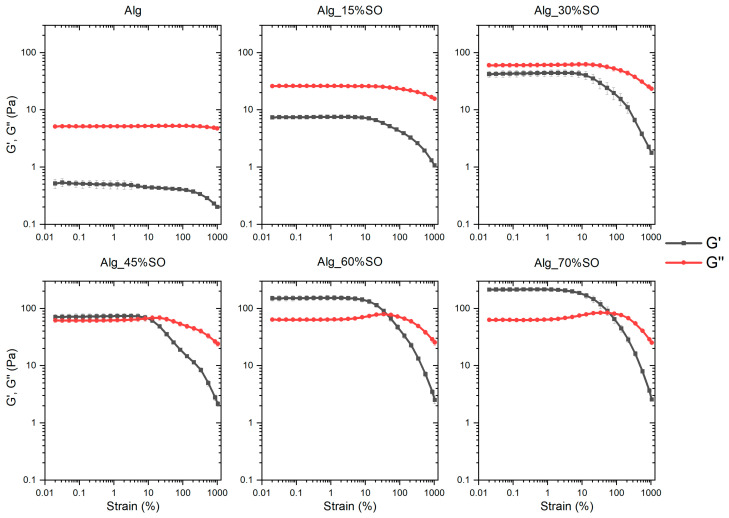
Amplitude sweep of emulsions prepared with different contents of SO. Alg: Sodium alginate solution; SO: Soybean oil.

An analysis of the influence of SO content on the rheological properties of the emulsified lipid network was carried out to assess the stability and consistency of the emulsion. The amplitude sweep responses of the samples are presented in Figure 1. The mixture before gelation exhibited the typical rheological behavior of emulsions. When the dispersed SO was a low fraction, the system was a viscous liquid as the G″ in the linear range was much higher than G′, and this characteristic held up until the SO reached a 30% oil content. Across all samples, the elasticity (G′) of the system in the linear region was significantly increased with the continuous addition of the SO, as higher mechanical energy was required to deform higher quantities of oil droplets and higher surface area [23]. In parallel, the value of G″ in the linear region also showed a significant increase when SO reached 30% but kept constant from 30% to 70%. This was because the collision between droplets did not impede the flow at low concentrations until the oil fraction reached a value of 30%. With an increase in both G′ and G″ with SO content, a cross-over point (where G′ = G″) was observed in the samples Alg_45%SO, Alg_60%SO and Alg_70%SO. Furthermore, the cross-over strain increased with the SO content. This indicated a higher flexibility of the emulsions when a strain was applied. In all samples, G′ and G″ were constant at low strain, then decreased as the strain amplitude increased, due to the irreversible deformation of the sample. All samples had a linear range up below 3% of strain, allowing accurate testing for frequency sweep tests.

**Figure 2:**
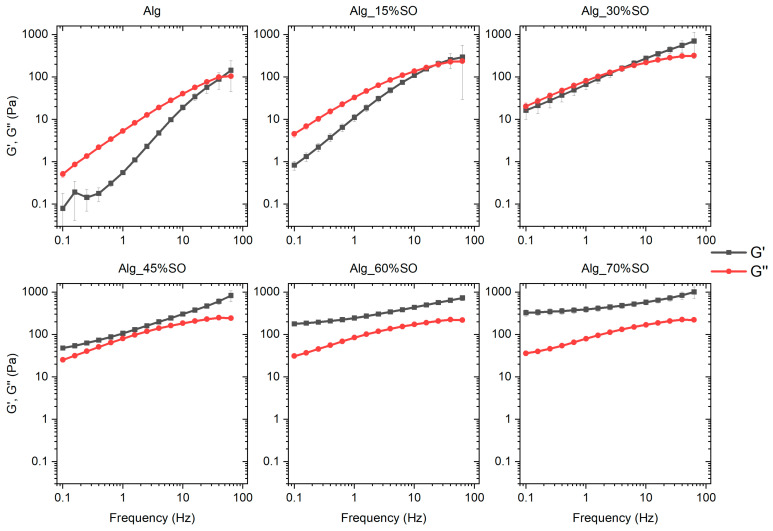
Frequency sweep of emulsions prepared with different contents of SO. Alg: Sodium alginate solution; SO: Soybean oil.

A frequency sweep was conducted at 0.1% strain, which was low enough to ensure that all the systems would be in the viscoelastic region and not disrupted by the strong shear; results are shown in Figure 2. Due to the limit of the equipment, the values of G′ and G″ measured at extreme low frequencies (0.01 to 0.1 Hz) were too noisy and therefore are not presented. This is also the reason why a few of the data showed a large deviation in Alg. Like the amplitude sweep results, the system transformed from a predominantly viscous liquid (G″ > G′) to an elastic gel (G′ > G″) with a weak dependence on frequency for the sample having a higher content of SO at the frequency range 0.1 to 10 Hz, at a critical weight fraction between 30% and 45%. This transition from a viscous liquid to a gel-like state as a function of entrapped oil was also reported in soybean protein- and methylcellulose-stabilized emulsions, justified by a higher degree of oil packing [24,25], as can be confirmed by the confocal images in the current work. At high frequencies, all the samples eventually showed elastic nature (G′ > G″) because the relaxation times of these samples were higher than the inverse of the frequency, which is a typical behavior of elastic materials.

**Figure 3:**
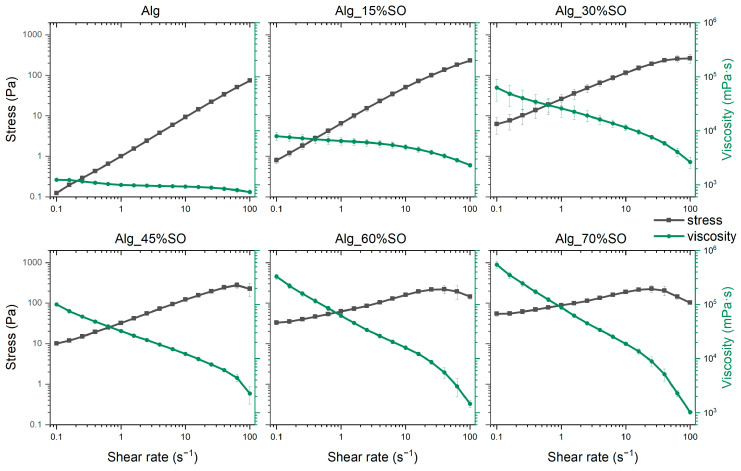
Flow curves of emulsions containing different contents of SO. Alg: Sodium alginate solution; SO: Soybean oil.

The flow curves of the emulsions are illustrated in Figure 3, which show a shear thinning behavior in all the samples. To quantitatively describe the change of the flow characteristics, data were fitted with the power-law model [26]:

(1) $${\sigma =K\dot{\gamma }}^{n}$$

where *σ* is the shear stress (Pa), *K* the consistency index (Pa·s^n^), and *n* the flow index. Fitted parameters are shown in Table 1. It is worth mentioning that there was a decrease in stress at high shear rate (>30 s^−1^) in samples Alg_45%, Alg_60%, and Alg_70%, and this critical shear rate became lower as the SO content became greater. This is attributed to the destruction of the structure due to high shear or the slippage between the sample and the geometry. These data were discarded to perform the model fitting.

As for the shear thinning behavior, the observed reduction in viscosity could be attributed to the alignment of particles in the direction of flow and the disruption of the gel network in the concentrated emulsions subjected to shear forces [27]. In addition, at low shear rate (<10 s^−1^), samples containing a higher amount of SO have higher viscosity, also indicated by a higher *K* value. When the oil fraction was high, the droplets were in closer proximity to each other, leading to a stronger interaction and hindered movement [28]. However, this effect was much less prominent in the high shear regime because droplets were deformed and aligned. At the highest tested shear rate (100 s^−1^), all samples showed similar viscosity, indicating that the predominant factor influencing the flow behavior was the alginate, instead of oil droplet interactions.

### 2.2. Color Measurement of Gelled Emulsions

The color of foods is the first attribute that consumers perceive, and plays a vital role in the acceptability of meat products [29]. The marbling of beef is mainly composed of fat tissues that has a white-to-yellow color [30]. Therefore, the color values (L, a and b) of gelled SO were measured and compared with the real beef fat trim. The color of the Alg gel was not recorded due to its transparent nature. As shown in Table 2, the fat trim had a light-yellow color value. The lightness (L) of gelled samples increased significantly as a function of SO addition, probably due to the formation of a denser emulsion, as droplets might scatter light more intensely [13]. All the samples showed close-to-zero redness (a) values. The slightly higher redness (a) value of beef fat trimming was likely to have originated from the myoglobin of some remaining muscle tissue. The yellowness (b) increased with SO content mainly because the pea protein and the SO are yellow. As a result, the color difference (ΔE) between the gelled SO and real fat trimming became smaller as a function of SO content, reaching a best value of 5.23. In comparison, the just-noticeable color difference was 2.3 [31]. Therefore, the formula could be used for substituting animal fat in terms of color, but it could further benefit from adding a greater amount of pea protein or colorant for an optimized appearance. Pictures of the emulsion gels and beef fat trimming are shown in Figure S1 in the Supplementary Materials.

### 2.3. Confocal Laser Scanning Microscopy

**Figure 4:**
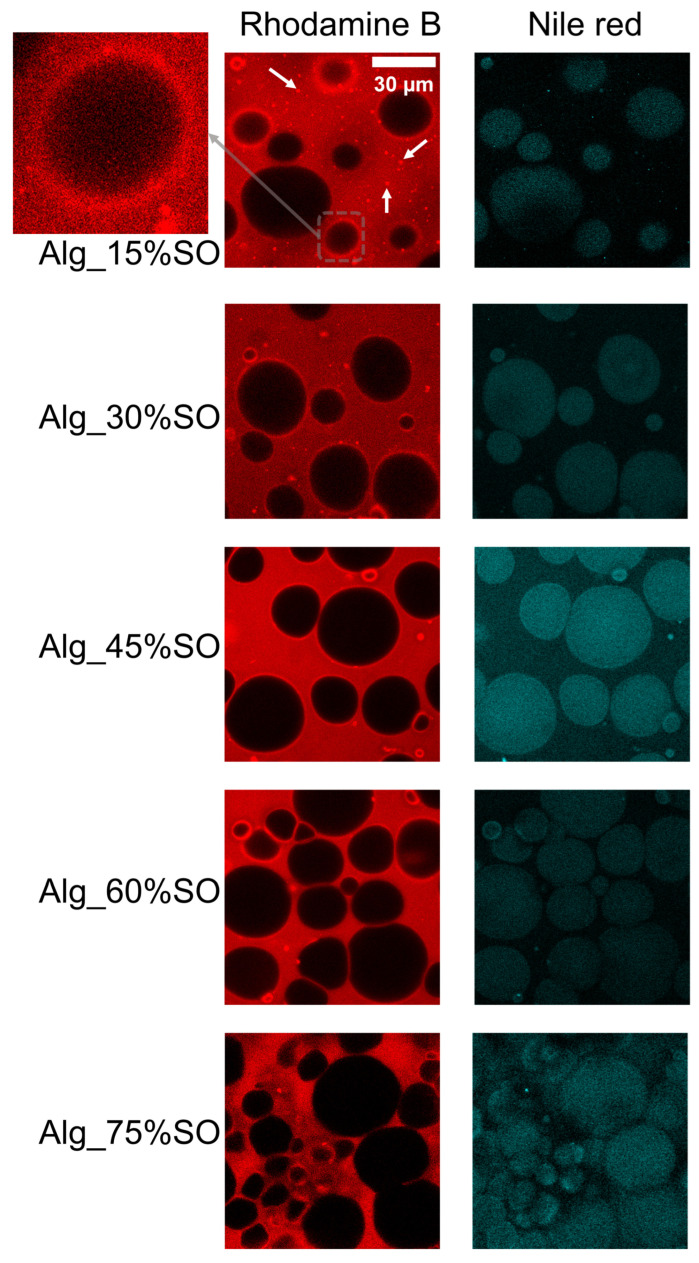
Protein and lipid distribution of gelled emulsions with different SO contents. Stained protein (Rhodamine B) is shown as red and lipids (Nile red) are shown as a cyan color. White arrows represented protein aggregations. One droplet in Alg_15%SO is zoomed (arbitrary scale) on the left side, showing a protein rim around it. The scale bar was 30 μm. Alg: Sodium alginate solution; SO: Soybean oil.

The distribution of both proteins and lipids was visible in the confocal microscopy images. As shown in Figure 4, lipids appeared predominantly as spherical droplets in Alg_15%SO, Alg_30%SO, and Alg_45%SO, and became more densely packed and with a polyhedral form in Alg_60%SO and Alg_70%SO. With increasing SO content, the number of smaller droplets increased due to the increase in the internal phase fraction that increased droplet collision [32]. Rhodamine B-stained images revealed highlighted rims around the droplets, indicating a higher concentration of protein that was able stabilize the oil droplet structure. The rims tended to appear in lower SO content samples and around smaller droplets, which might be due to the higher surface tension required for smaller droplets. A similar phenomenon of interfacial protein films was also reported in emulsions stabilized by rapeseed protein [33]. Additionally, protein aggregations appeared as speckles in the rhodamine B-stained images (white arrows). The trend that the amount of protein aggregation decreased with increasing SO content resulted in a better protein dispersion dissolution due to increased mechanical stirring and stronger interfacial protein requirements.

### 2.4. Differential Scanning Calorimetry (DSC)

**Figure 5:**
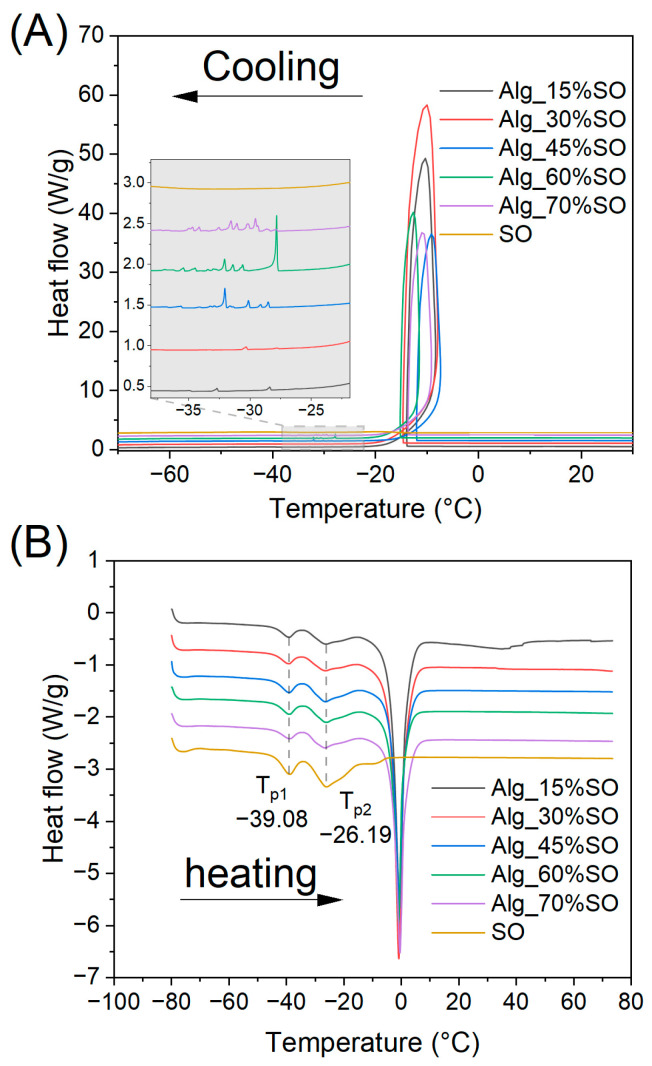
Differential scanning calorimetry (DSC) curves of gelled emulsions prepared with different SO during (**A**) cooling and (**B**) melting. Data from different samples were offset for readability, and the detailed cooling curve is zoomed between −23 and −37 °C and presented as an inset in (**A**). Peak temperatures in the melting curves are annotated. Alg: Sodium alginate solution; SO: Soybean oil.

The DSC crystallization curves of the gels are presented in Figure 5. In the cooling curve, there was an obvious loop-shaped peak present in all samples containing water, which corresponded to the crystallization of supercooled unbound water. The crystallization of water would release a significant amount of energy in a short time, increasing the temperature of the samples [34,35]. Most of the crystallization of lipids was assumed to also take place starting at the same onset temperature as the pure SO of around −14.6 °C, but the signal overlapped with the water crystallization. Moreover, it appears that there were some random small peaks in the crystallization curve in the gelled samples containing SO ranging between −27 °C and −37 °C (zoomed in Figure 5A). The position, peak height, and peak area were not consistent across replicates or even the sample that received two consecutive runs (data not shown). However, it followed the general trend that the higher the SO fraction in the sample, the higher was the crystallization peak. A similar phenomenon of multiple small peaks was also reported in water-in-oil emulsions, whereas emulsions containing a higher water fraction had fewer peaks [36]. The phenomenon was explained by differences in the droplet size distribution. In our case, such an observation could potentially result from the emulsification of the lipids and the formation of ice crystals. Unlike the free SO, the lipids in the emulsion were confined by the droplets, thus their molecular mobility was impaired, causing a lower solidification temperature [37]. In addition, ice crystals might physically damage the gel matrix, leading to lipid leaching, and those leached lipids may crystalize rapidly outside the alginate matrix. In parallel, it is possible that these peaks could also be a result of a melting-recrystallization process as the lipid recrystallization could take place even in a short heating period [38] enhanced by the mentioned water crystallization. 

Due to the partial overlapping of lipid and water crystallization and melting, only the onset and peak temperatures of the lipid endothermic curves during heating were identified and compared. The resulting thermal characteristics are summarized in Table 3. Two melting peaks at around −39.1 °C and −26.3 °C were identified, representing a lipid crystal polymorphism with different stability. The thermal transition temperatures of lipids melting in SO were similar to those previously reported [39]. As shown in Figure 5A and Table 3, the melting onset and two peak temperatures were constant across all the samples, indicating that thermal treatment had a consistent effect on the lipids regardless of their emulsified status. The melting curves again confirmed the formation of lipid crystals at the mentioned temperatures masked by ice crystallization. Meanwhile, the findings also indicated that the lipids in a protein-emulsified alginate gel system would not be changed due to the emulsification or gelation process. 

### 2.5. Fourier Transform Infrared (FTIR) Spectroscopy

**Figure 6:**
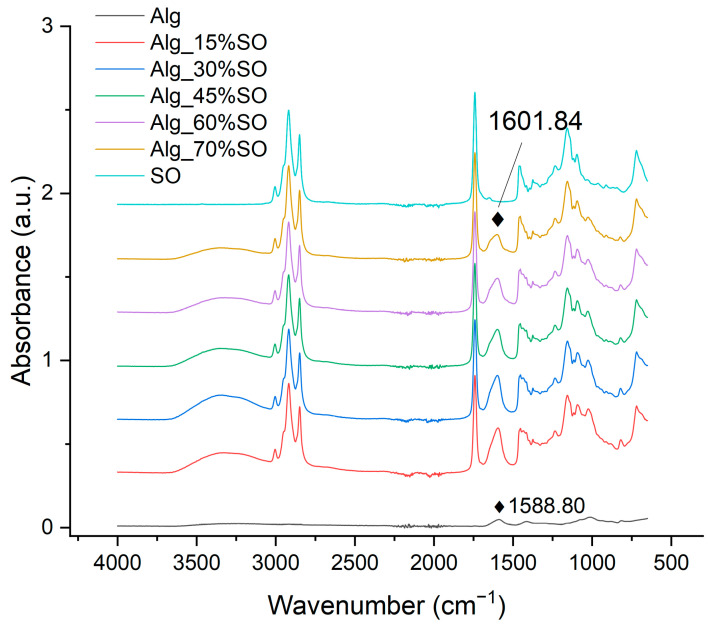
FTIR spectra of alginate (Alg), emulsions, and pure soybean oil (SO). Alg: Sodium alginate solution; SO: Soybean oil.

FTIR spectra can reflect functional group changes as a function of the SO content in the gelled emulsion. The spectra of alginate-gelled SO and pure SO are presented in Figure 6. Alginate had a relatively low signal due to the rigidity of the molecules after freeze drying and the small contact area with the ATR crystal. It exhibited two peaks due to carbonyl vibrations of the carboxylate group: at 1588 cm^−1^ and 1415 cm^−1^ due to anti-symmetric and symmetric stretches, respectively [40]. The spectral region from 1080 cm^−1^ to 951 cm^−1^ is assigned to the stretching vibration of the C–O bond, as well as the deformation of the C–C–H and C–O–H bonds [41]. In SO, the characteristic peaks at 2918 cm^−1^ and 2850 cm^−1^ reflected the -CH_2_ symmetric and anti-symmetric stretching vibrations, and the peak at 1741 cm^−1^ was attributed to the C=O stretching of triglycerides [42]. Peaks at 3006, 1650, and 1097 cm^−1^ were caused by CH=CH, -C=C-, and -C-O-C- groups, respectively, indicating an abundant amount of unsaturated fatty acids [43]. 

Although all the gelled samples containing SO were freeze dried, there was a broad spectrum corresponding to O-H stretching from water at 3350 cm^−1^. This could likely be due to the water-holding capacity of pea protein and alginate in the sample that increased the hydrogen bonding in the gel [44]. In general, the spectra of gelled samples showed a gradual transition from alginate to SO, without the formation of any new peaks that were not present in SO or alginate. However, there was a slight peak shift from 1588.8 cm^−1^ in Alg to a higher wavenumber as a function of SO content, reaching 1601.8 cm^−1^ in the Alg_70%SO sample. As no peak was identifiable in SO in that range, the shift could be assigned to the interaction between alginate and pea protein. Previous research also supported the complex-forming ability between globular protein and alginate [45]. In terms of pea protein, however, the typical conformation-sensitive amide I, amide II or amide III groups were not identifiable in the gelled samples. The strongest amide I group signal is reported to be around 1650 cm^−1^, caused by C=O stretching [46], but it might be overlapped by the cis C=C or C=O stretching from SO at 1650 cm^−1^ or H–O–H bending of water at around 1645 cm^−1^ in this case [47]. The absence of pea protein amide groups per se was mostly possible due to its low concentration. As for lipids, the peaks showed no shifts, indicating negligible functional group alteration in the emulsification and gelation processes, which have been reported in other oil and alginate emulsions [48]. 

### 2.6. Surface Extractable Lipid

**Figure 7:**
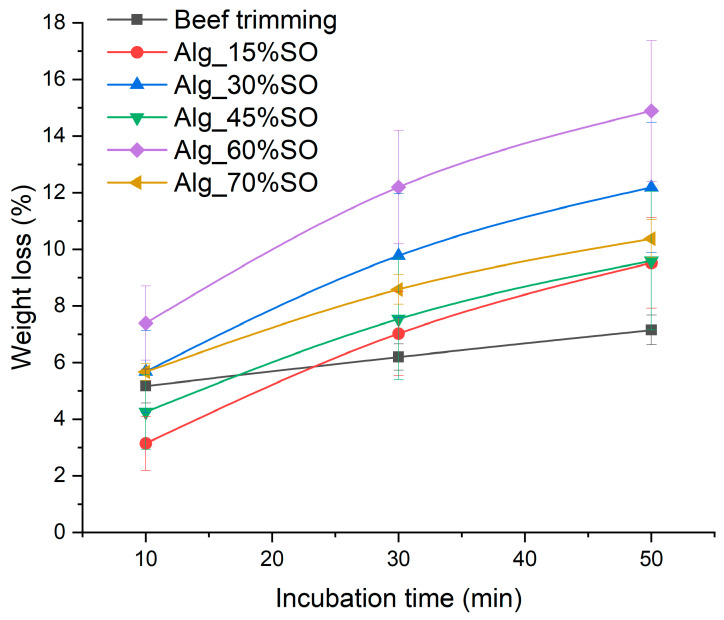
Weight loss of gelled emulsions mixed in hexane as a function of incubation time. Alg: Sodium alginate solution; SO: Soybean oil.

The extraction rate and amount of extractable lipid by an organic solvent from a gel can reflect the structural integrity of the gel system, which may indicate stability against oil leaking. The percentages of extractable lipid from the gels and beef fat trimming are presented in Figure 7. It is evident that weight loss continued to increase with extraction time up to 50 min, but even the highest value was less than 20%. This indicates that the solvent gradually infiltrated the sample and released entrapped lipid into the solvent. Meanwhile, such extraction was much slower than the system composed of gelled butter structured by alginate and milk protein, as shown in a previous work. This is likely due to the better emulsifying capacity of pea protein isolate than the natural protein present in butter. 

Overall, the structural stability is a result of calcium-induced alginate networking [49]. After a short incubation time (10 min), Alg_15% showed significantly lower weight loss, due to a lower oil content and a higher alginate matrix volume. After prolonged solvent extraction (>30 min), all gelled emulsions showed a non-significant difference among themselves (*p* > 0.05), but slightly greater lipid loss than the beef fat trimming, indicating greater gel permeability than the native animal collagen network and adipose cells. The extractability of the lipids did not show a significant correlation with the oil content after longer extraction, which differs from other alginate-based systems where hydrophobic fillers tend to separate from the gel at higher concentrations [50]. This indicates the compact matrix structure of the hydrogel network, probably due to the high incompatibility between pea protein and hexane, which impedes solvent penetration. In addition, the constant ratio between protein content and SO content might contribute to maintaining the integrity of the structure when heated extraction solvent was present. This characteristic is favorable for practical production, as similar heat stability can be achieved without concern for extreme high or low oil content that may influence the product.

## 3. Conclusions

In the present study, a completely plant-based fat analog was developed based on a two-step gelation method with sodium alginate, soybean oil, and pea protein. The pre-gelled emulsion exhibited a conversion from a viscous fluid to a more viscoelastic material with increasing oil fraction. After gelation, the color of the product was light yellow, similar to that of real beef fat trimming. However, a slightly greater yellowness could be a key factor in better mimicking the appearance of animal fat tissue. During the production of the gelled emulsion, the addition of pea protein stabilized the soybean oil and protected it from heat-induced leaching by the gelled alginate matrix, which simulated the slow rendering effects of animal fat trims when heated, overcoming the challenge of fast melting of solid fat from plant sources. However, further studies are required to investigate the lipid-releasing behavior at higher cooking temperatures. Furthermore, vibrational spectra and differential scanning calorimetry results revealed no detectable chemical modification or alteration of the melting curve of the entrapped lipids, further confirming the safety of this product. This study presents a novel approach to structuring liquid plant-based fat that can be easily scaled up. In the future, relative studies on texture profile and sensory trials, particularly when the gel is incorporated with the protein portion of plant-based meat analogs, would be advantageous.

## 4. Materials and Methods

### 4.1. Materials

Soybean oil (SO) was purchased from a local grocery store. Pea protein isolate was obtained from ADM (Decatur, IL, USA). Food grade sodium alginate (Alg) was purchased from Landor Trading Co., Ltd. (Montréal, QC, Canada). Nile red and rhodamine B were purchased from Thermo Fisher Scientific (Waltham, MA USA). Beef brisket fat trimming was cut from a fresh carcass and stored at −18 °C before use.

### 4.2. Pea Protein Isolate Purity Determination

The purity of the commercial pea protein isolate was determined using a nitrogen analyzer (Elementar, Long Island, NY, USA). A conversion factor of 6.25 was adopted [51]. Pea protein isolate purity was 76% and it was used for formulation calculations.

### 4.3. Preparation of the Gel

The emulsion was prepared by a series of specific mixing steps. First, 3% (*w/w*) sodium alginate powder was dissolved in deionized water at 70 °C with stirring, then cooled and stored at 20 °C for at least 24 h for complete dissolution. Then, the alginate solution and SO were separately water bathed at 70 °C. Pea protein isolate was dissolved in the heated alginate solution at 1% (*w*/*w*) of the intended mass of SO. SO was then added gradually to the alginate solution containing pea protein: each time 5 g of SO was transferred, the mixture underwent thorough stirring until a creamy texture and smooth surface was achieved; the addition of SO and the stirring were repeated till the target amount of SO was reached. The emulsion was then gelled with 1.5% CaCl_2_ solution by either extruding the emulsion from a piping bag to the CaCl_2_ solution (for FTIR) or adding CaCl_2_ solution to the centrifuge tubes containing the emulsion. Gels were prepared with different amount of SO and their compositions are summarized in Table 4. The upper limit of SO was chosen as 70% because a higher amount resulted in a collapse of the emulsion in which the excess amount of SO would form a separate phase that could not be incorporated via stirring of the emulsion.

### 4.4. Rheology

Rheology was used to test pre-gelled emulsions to characterize the flow characteristics of the system as a function of oil content with a Discovery HR-3 rheometer (TA Instruments, New Castle, DE, USA). Samples were loaded onto a hatched Peltier plate, and a 40 mm diameter hatched plate upper geometry was used to minimize slippage. 1000 μm was set as the running gap with a trim gap of 1050 μm. An amplitude sweep performed from 0.02% to 1000% strain at 1 Hz of frequency was first conducted to determine the linear range prior to other small amplitude oscillation tests. The frequency sweep was set from 0.01 to 100 Hz at 0.1% strain, and the apparent viscosity was recorded using a flow sweep conducted from 0.1 to 100 s^−1^ to mimic various real processing conditions. All tests were conducted at 50 °C (equilibrium for 3 min prior to tests) with a Solvent Trap to reduce water evaporation for at least three replicates. Data were collected using TRIOS software (TA Instruments).

### 4.5. Colorimetry

The lightness (*L*), redness (*a*), and yellowness (*b*) of gelled SO, pea protein isolate, and beef fat trimmings were recorded with a Chroma meter CR-400 portable colorimeter (Konica Minolta Sensing Americas, Inc., Ramsey, NJ, USA). The illuminant was D65 and the measurement area was 8 mm diameter. The calibration plate has values of *L* = 94.13, *a* = −0.20, and *b* = 3.19. Five measurements for each sample were conducted. Color differences (Δ*E*) were calculated between the color values of the gelled sample or pea protein and that of beef fat trimming using the CIE76 method:

(2) $$\Delta E=\sqrt{{\left(L_{sample}-L_{trim}\right)}^{2}+{\left(a_{sample}-a_{trim}\right)}^{2}+{\left(b_{sample}-b_{trim}\right)}^{2}}$$

### 4.6. Confocal Laser Scanning Microscopy

Nile red and rhodamine B were used to stain lipids and proteins, respectively. Nile red (2 mg/mL in acetone) was first added to SO (1%, *v*/*v*). Then, the pre-gelled emulsion was prepared as described with the stained SO. Around 0.3 g emulsion was extracted from the system and placed on a curvature slide. 5 μL of rhodamine B (2 mg/mL in deionized water) was then spiked and mixed with the emulsion. 100 μL of 1.5% CaCl_2_ solution was dropped to form the gel. A coverslip was immediately placed and gently flattened into place, and the excess CaCl_2_ solution was removed using a filter paper. The slides were sealed with transparent nail polish and stored in the dark at 4 °C prior to confocal microscopy imaging.

A Zeiss LSM900 Confocal Microscope (Oberkochen, Germany) was used to capture the images. Excitation wavelengths were 488 nm and 568 nm for Nile red and rhodamine B, respectively. Images were post-processed with ImageJ software (Laboratory for Optical and Computational Instrumentation, Madison, WI, USA).

### 4.7. Differential Scanning Calorimetry (DSC)

The crystallization and melting profiles of the free and bound SO were determined using a DSC2500 (TA Instruments). 10 mg of gelled sample or SO was extracted to a Tzero aluminum pan for the test. The samples were first equilibrated at 35 °C for 3 min to eliminate any thermal memory. Then, they were cooled to −80 °C at 10 °C/min, stood for 5 min for complete crystallization and finally heated again to 80 °C at 10 °C/min. An empty pan was used as the reference. TRIOS software (TA Instruments) was used to collect and process data to determine the onset and peak temperatures.

### 4.8. Fourier Transform Infrared (FTIR) Spectroscopy

The pre-gelled emulsion was prepared as described before. Then, it was extruded via piping bags into constantly stirred 1.5% CaCl_2_ solution to form pipe-shaped gels. The system was allowed to stand in the CaCl_2_ solution for over 24 h to ensure full gelation. Then, the gel was freeze-dried to remove excess water. A portable FTIR device coupled with a triple-reflection Attenuated Total Reflectance (ATR) sampler (Agilent Technologies, Santa Clara, CA, USA) was used for the test. The scanning wavelength range was from 4000 to 650 cm^−1^ with a resolution of 4 cm^−1^. Sixty-four scans were performed to increase the signal. Ethanol was used to clean the sampling area and a background spectrum collection was performed between each test. A sample press was used to maximize the contact between the dried sample and the sampling crystal. Triplicate tests were conducted for each sample. Agilent MicroLab PC software was used for data collection.

### 4.9. Surface Extractable Fat

The stability of the gel against leaking upon heat was determined with a modified hexane extraction method [52]. Briefly, 5 g of gel was weighed and immersed in heated hexane (50 °C). After up to 50 min, the gel was removed from the hexane, and the excess hexane was removed with a filter paper. The weight was recorded as a function of immersion time. Beef fat trimming was used as control.

### 4.10. Statistical Analysis

Statistical analysis was performed using Origin Pro (Origin Lab, Northampton, MA, USA). Analysis of variance (ANOVA) was conducted to compare the differences between groups followed by a Tukey test with *p* < 0.05 as the significant level.

## Figures and Tables

**Table 1:** The fitted parameters of the power-law model of emulsions with different amounts of SO.

	n	K	R^2^
Alg	0.936 ± 0.004	1.043 ± 2.325	0.9997
Alg_15%SO	0.844 ± 0.015	6.272 ± 2.387	0.9956
Alg_30%SO	0.590 ± 0.017	25.421 ± 2.400	0.9882
Alg_45%SO	0.542 ± 0.007	33.083 ± 2.336	0.9981
Alg_60%SO	0.352 ± 0.010	65.902 ± 2.348	0.9906
Alg_70%SO	0.282 ± 0.010	92.449 ± 2.342	0.9875

Results shown as estimate ± standard error.

**Table 2:** Color values of gelled SO, pea protein, and beef fat trimming.

Sample	L	a	b	ΔE
Alg_15%SO	50.26 ± 2.67 ^A^	−0.17 ± 0.20 ^A^	0.85 ± 1.18 ^A^	28.82 ± 2.58 ^A^
Alg_30%SO	58.08 ± 1.17 ^B^	−0.13 ± 0.25 ^A^	2.48 ± 0.25 ^AB^	20.9 ± 1.02 ^B^
Alg_45%SO	65.09 ± 1.11 ^C^	−0.40 ± 0.20 ^A^	3.96 ± 0.10 ^BC^	13.92 ± 1.06 ^C^
Alg_60%SO	69.89 ± 1.97 ^D^	−0.55 ± 0.04 ^A^	5.15 ± 0.27 ^CD^	9.28 ± 1.76 ^D^
Alg_70%SO	75.94 ± 3.77 ^E^	−0.53 ± 0.51 ^A^	6.62 ± 0.98 ^D^	5.23 ± 1.75 ^E^
Pea protein	89.08 ± 0.23 ^F^	1.73 ± 0.05 ^B^	16.00 ± 0.31 ^F^	13.27 ± 0.15 ^C^
Beef fat trimming	77.72 ± 2.71 ^E^	2.28 ± 0.67 ^B^	9.16 ± 1.52 ^E^	-

Results shown as mean ± standard deviation; different superscript letters represent significantly different result according to Tukey’s test (*p* < 0.05).

**Table 3:** Melting characteristic temperature of gelled emulsion and pure soybean oil.

Sample	T_on_	T_p1_	T_p2_
Alg_15%SO	−43.82 ± 0.21 ^A^	−39.09 ± 0.14 ^A^	−26.18 ± 0.14 ^A^
Alg_30%SO	−43.96 ± 0.28 ^A^	−39.19 ± 0.28 ^A^	−26.33 ± 0.29 ^A^
Alg_45%SO	−44.15 ± 0.18 ^A^	−39.09 ± 0.11 ^A^	−26.43 ± 0.27 ^A^
Alg_60%SO	−43.74 ± 0.55 ^A^	−39.10 ± 0.18 ^A^	−26.46 ± 0.15 ^A^
Alg_70%SO	−43.79 ± 0.23 ^A^	−39.07 ± 0.23 ^A^	−26.58 ± 0.21 ^A^
SO	−44.18 ± 0.04 ^A^	−39.09 ± 0.01 ^A^	−26.28 ± 0.13 ^A^

T_on_: onset temperature of the first peak, T_p1_: the peak temperature of first endothermic peak, T_p2_: the peak temperature of second endothermic peak. Results shown as mean ± standard deviation; same superscript letter represents non-significant differences in a Tukey test (*p* > 0.05).

**Table 4:** Sample codes and emulsion formulations.

Sample Code	Alginate Solution (g)	Pea Protein (g)	SO (g)
Alg	100	0	0
Alg_15%SO	85	0.15	15
Alg_30%SO	70	0.3	30
Alg_45%SO	55	0.45	45
Alg_60%SO	40	0.6	60
Alg_70%SO	30	0.7	70

## Data Availability

The data presented in this study are available on request from the corresponding author.

## Supplementary Materials

The following supporting information can be downloaded at: https://www.mdpi.com/article/10.3390/gels9050393/s1, Figure S1: Gelled emulsions (left) and beef fat trimming.
