# Supplementary material for: A Plant-Based Animal Fat Analog Produced by an Emulsion Gel of Alginate and Pea Protein
Source: Gels. 2023 May 9;9(5):393. doi: 10.3390/gels9050393 (PMC10217620; doi:10.3390/gels9050393)
Supplement: Supplementary file 1 [file gels-09-00393-s001.zip › gels-2365863 supplementary material.pdf]

gels MDPI

Supplementary Material

A Plant-Based Animal Fat Analog Produced by an Emulsion Gel of Alginate and Pea Protein

Chong Teng and Osvaldo H. Campanella *

Department of Food Science and Technology, Ohio State University, 2015 Fyffe Road, Columbus, OH 43210, USA; teng.165@buckeyemail.osu.edu
* Correspondence: campanella.20@osu.edu; Tel.: +1-6142477646

Supplementary Information

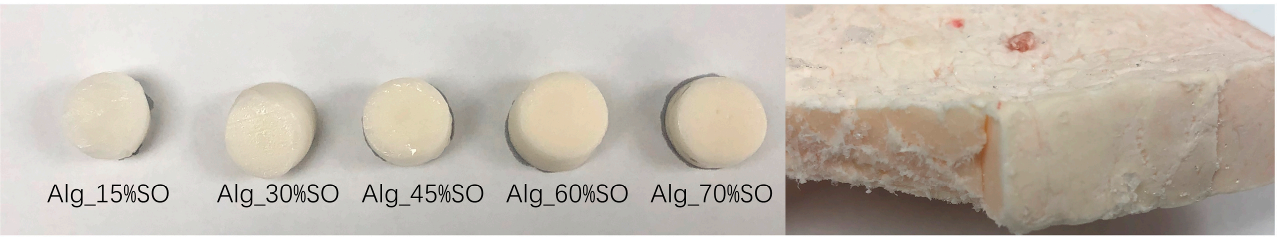

Figure S1. Gelled emulsions (left) and beef fat trimming.
